# Supplementary material for: An electrostatic switching mechanism to control the lipid transfer activity of Osh6p
Source: Nat Commun. 2019 Sep 2;10:3926. doi: 10.1038/s41467-019-11780-y (PMC6718676; doi:10.1038/s41467-019-11780-y)
Supplement: Supplementary file 1 — Supplementary Information [file 41467_2019_11780_MOESM1_ESM.pdf]

Supplementary Information

An electrostatic switching mechanism to control the lipid transfer activity of Osh6p

Lipp et al.

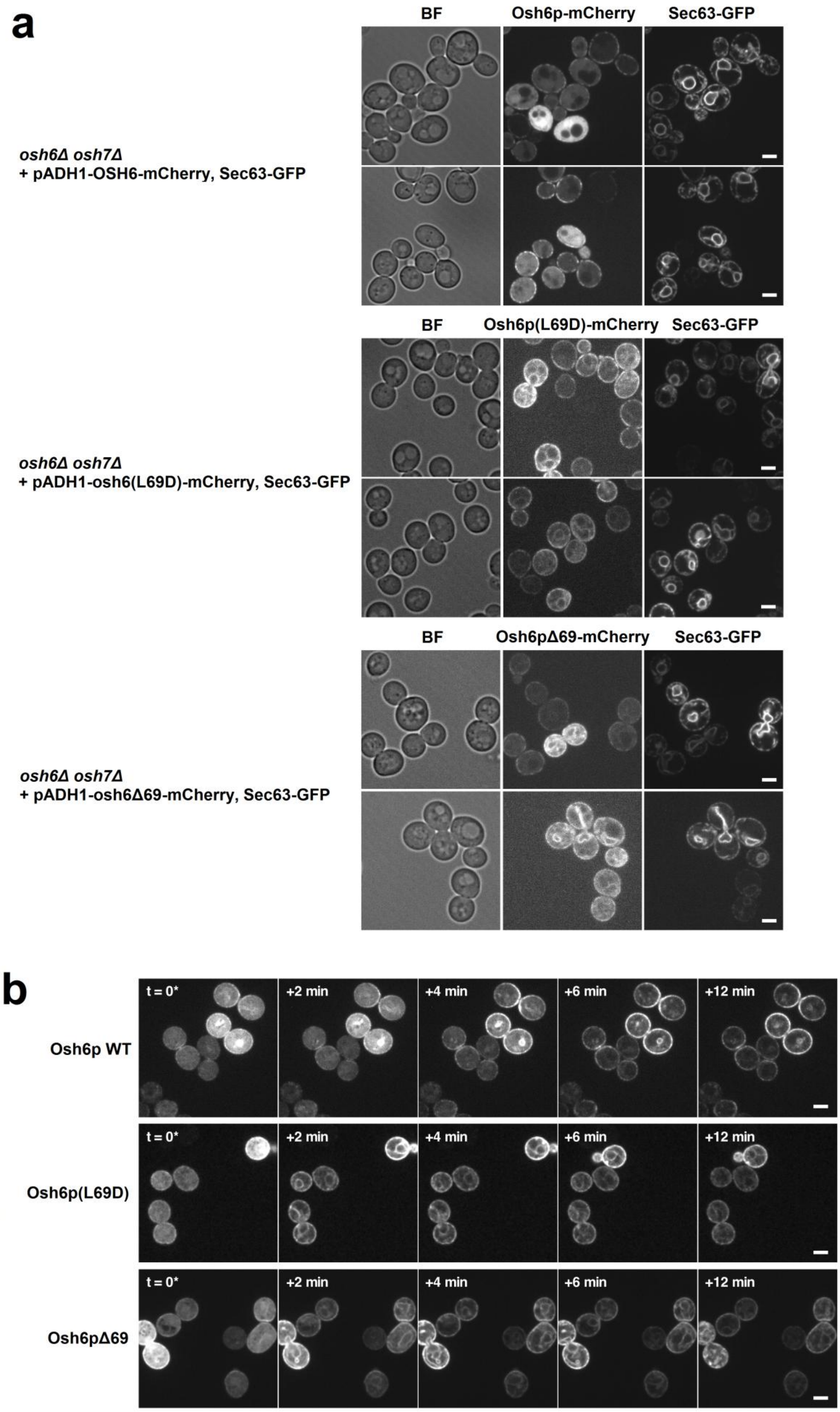
a
BF
Osh6p-mCherry
Sec63-GFP
osh6Δ osh7Δ
+ pADH1-OSH6-mCherry, Sec63-GFP
BF
Osh6p(L69D)-mCherry
Sec63-GFP
osh6Δ osh7Δ
+ pADH1-osh6(L69D)-mCherry, Sec63-GFP
BF
Osh6pΔ69-mCherry
Sec63-GFP
osh6Δ osh7Δ
+ pADH1-osh6Δ69-mCherry, Sec63-GFP
b
Osh6p WT
Osh6p(L69D)
Osh6pΔ69
t = 0*
+2 min
+4 min
+6 min
+12 min

Supplementary Figure 1. Cellular localization and PS transport activity of Osh6p wild-type and N-terminal mutants

(a) Colocalization between Osh6p variants (WT, L69D or Δ69 truncation mutant), fused to mCherry, and the ER marker Sec63-GFP in *osh6Δ osh7Δ* cells. Two fields of cells are shown for each strain, with left panels showing bright-field (BF) images. **(b)** Redistribution of the PS reporter $C2_{Lact}$-GFP in PS-depleted yeast cells (*cho1Δ osh6Δ osh7Δ*) expressing Osh6p-mCherry WT, L69D or Δ69 mutant after exogenous addition of 18:1 lyso-PS. Images were taken every 2 min as indicated, with the first panel (t = 0*) showing the last time-point before the onset of $C2_{Lact}$-GFP signal transition. The absolute timing of this time-point varied between experiments (between 15 and 25 min after lyso-PS injection) due to instability of the lyso-PS suspension [1]. Scale bar: 5 μm.

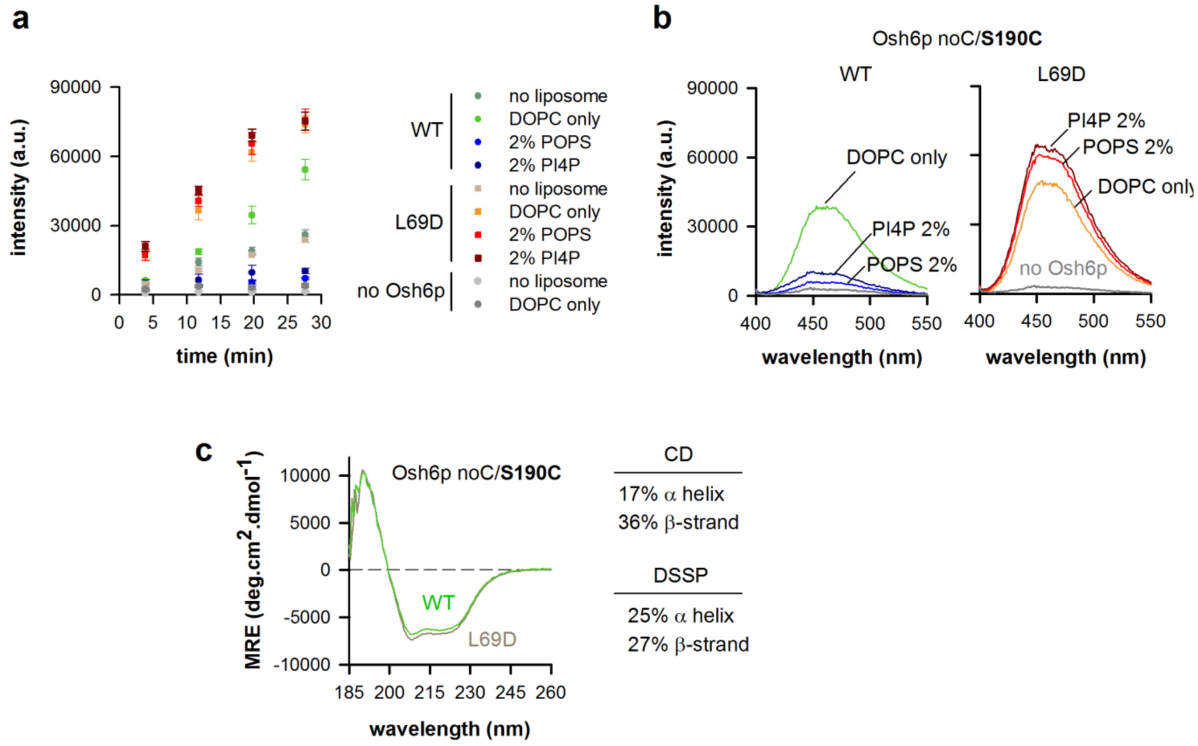

Supplementary Figure 2. Measure of the conformational state of Osh6p with an accessibility assay using CPM

(a) Time evolution of the CPM signal (λ_{ex}=387 nm) after adding the fluorophore (4 µM) to Osh6p noC/S190C or its L69D variant (400 nM) in HK buffer alone or in the presence of liposomes only made of DOPC (400 µM total lipids) or containing either 2 mol% POPS or PI4P. Control experiments are done without Osh6p protein. **(b)** Typical emission spectra of Osh6p noC/S190C and its L69D counterpart (400 nM) after an incubation of 30 min with 4 µM CPM in the presence of pure DOPC liposomes or liposomes doped with either 2 mol% POPS or PI4P. A control experiment is done without Osh6p protein (gray spectrum). **(c)** Far-UV CD spectrum of Osh6p noC/S190C and its L69D variant (10 µM) in 20 mM Tris, pH 7.4, 120 mM NaF buffer. The percentage of α-helix, β-sheet and turn, deriving from the analysis of the spectrum are given as well as the values deriving from the crystal structure (PDB ID: 4B2Z) using the DSSP algorithm. Source data are provided as a Source Data file.

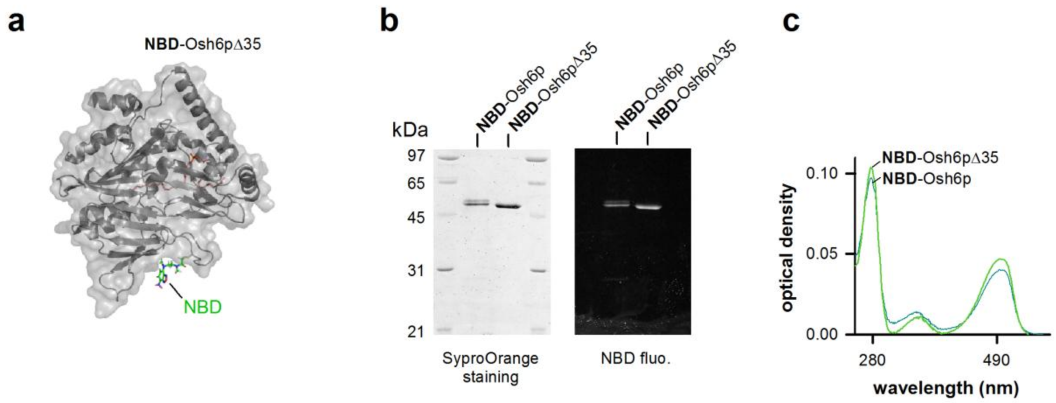

Supplementary Figure 3. Biochemical characterization of the NBD-Osh6p constructs
(a) Tridimensional model of the NBD-labeled Osh6pΔ35 based on the crystal structure of the protein (PDB ID: 4B2Z). Several cysteines (C62, C162 and C389) are mutated into serine; the threonine 262 in the β14-β15 loop is substituted into a cysteine. An N,N'-dimethyl-N-(acetyl)-N'-(7-nitrobenz-2-oxa-1,3-diazol-4-yl)ethylenediamine moiety (in stick, with carbon in green, nitrogen in blue and oxygen in red), manually built and energetically minimized, is grafted to the thiol function of the C262 residue. **(b)** SDS-PAGE of purified NBD-Osh6p WT and Δ35. The gel was directly visualized in a fluorescence imaging system to identify labeled proteins (right picture) and then stained with Sypro Orange to visualize the protein and molecular weight markers (left picture). **(c)** UV-visible absorption spectrum of the wild-type Osh6p and the Δ35 mutant labeled with NBD. Considering a purity grade of 100% for the protein, the analysis of the optical density at 280 nm (Trp) and 495 nm (IANBD) indicated that each construct was labeled with the probe in a 1:1 ratio. Source data are provided as a Source Data file.

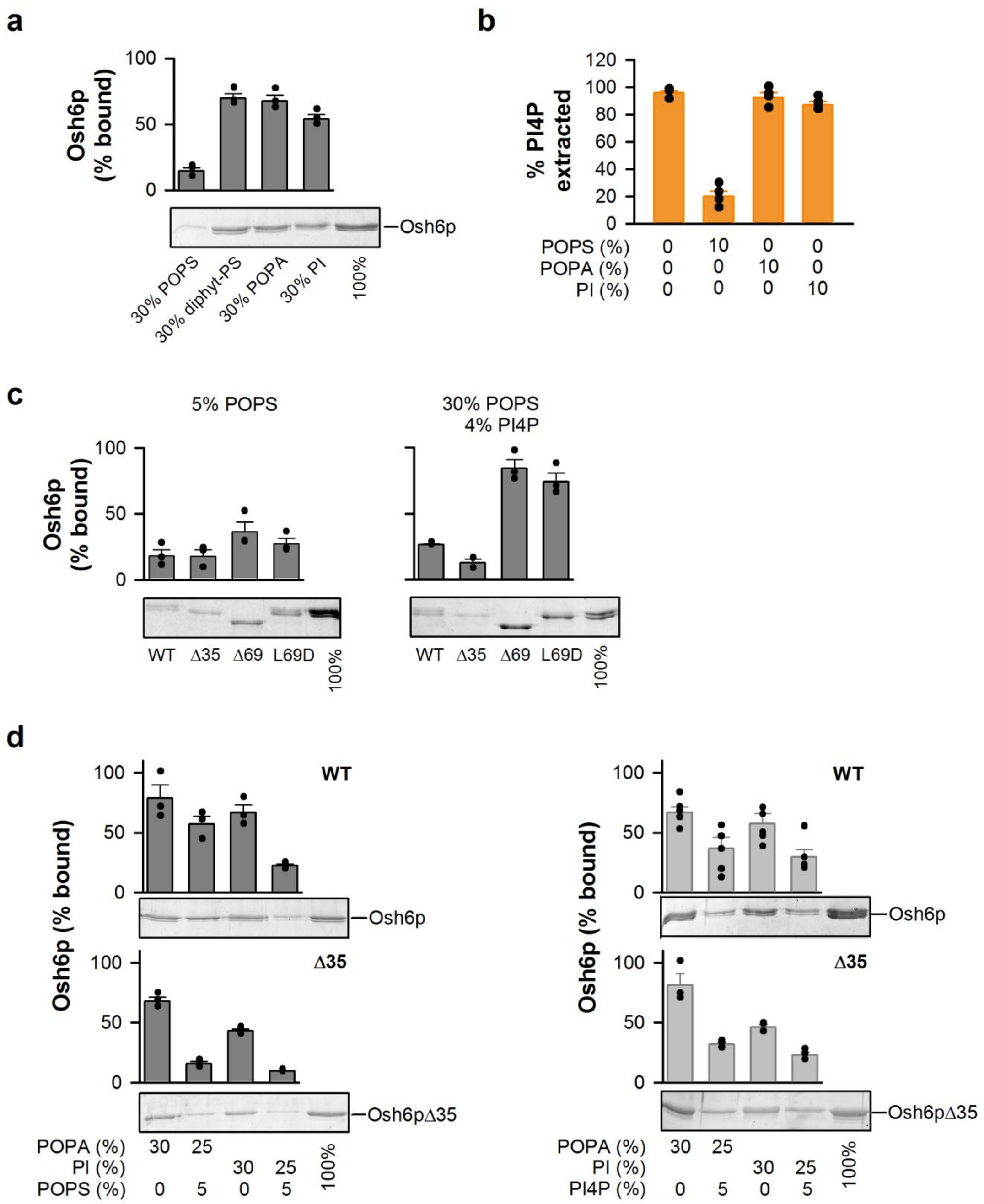

Supplementary Figure 4. Ligand-dependent binding of Osh6p to anionic membranes
(a) Flotation assay. Osh6p (750 nM) was incubated with DOPC liposomes (750 µM total lipids) enriched with 30 mol% of POPS, diphytanoyl-PS, POPA or liver PI (at the expense of DOPC). The bars correspond to the mean of three independent experiments. **(b)** PI4P extraction assay. NBD-PH$_{FAPP}$ (250 nM) was incubated with DOPC liposomes (80 µM lipids) containing 2 mol% diC16:0-PI4P or, additionally, 10 mol% of POPS, POPA or liver PI. The fluorescence spectra of the probe were measured (λ_{ex}= 460 nm) before and after adding Osh6p WT (3 µM). Reference spectra were recorded with liposomes devoid of PI4P. The percentage of extracted PI4P was calculated based on the fluorescence signal at 536 nm measured with and without Osh6p and with PI4P-free liposomes (n=4). **(c)** Flotation assay. Osh6p, Osh6pΔ35, Osh6pΔ69 or Osh6p(L69D) (750 nM) was mixed with DOPC liposomes (750 µM lipids) doped with 5 mol% of POPS or 30 mol% of POPS and 4 mol% of PI4P (n=3). **(d)** Flotation assay. Osh6p or Osh6pΔ35 (750 nM) was incubated with DOPC/POPA (7/3 mol/mol) or DOPC/PI (7/3) liposomes (750 µM lipids) containing or not 5 mol% of POPS or diC16:0-PI4P, at the expense of POPA or PI (n=3). Error bars correspond to s.e.m. Source data are provided as a Source Data file.

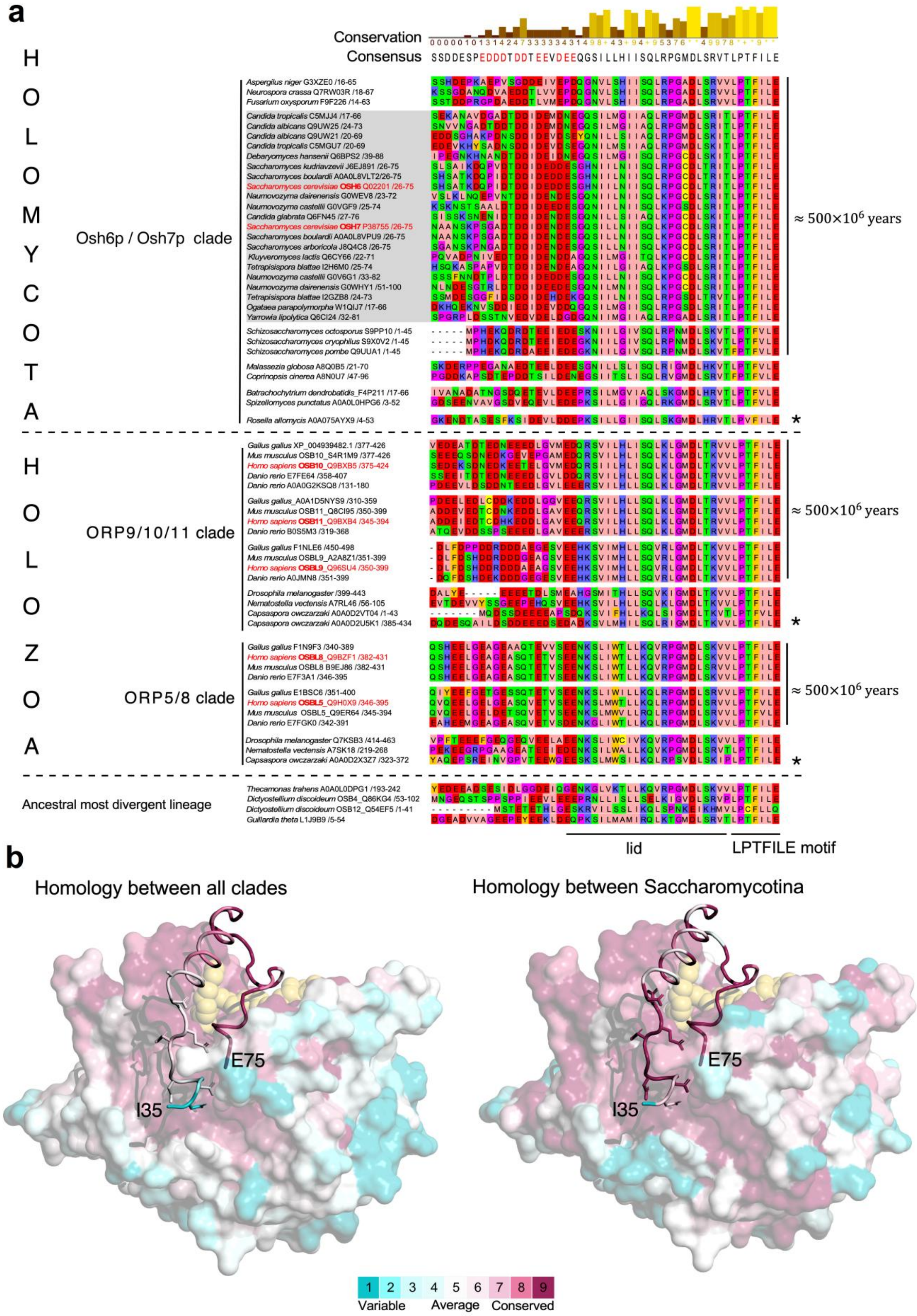
a
Conservation
Consensus
SSDDESPEDDDTDDTEEVDEEQGSILLHIISQLRPGMDLSRVTLPTFILE
HOLOMYCOTA
Osh6p / Osh7p clade
≈ 500×10⁶ years
HOLOZOA
ORP9/10/11 clade
ORP5/8 clade
Ancestral most divergent lineage
lid
LPTFILE motif
b
Homology between all clades
Homology between Saccharomycotina
E75
I35
1 2 3 4 5 6 7 8 9
Variable
Average
Conserved

Supplementary Figure 5. Conservation of an acidic stretch at the N-terminal end of the lid of putative PS/ PI4P exchangers

(a) Sequence alignment of putative PS/PI4P counter-exchangers containing the LPTFILE motif. The alignment procedure was performed on a set of 104 eukaryotes sequences from 73 eukaryotes species but for clarity, only 35 sequences of representative species are shown. Genera, species, UniprotKB accession number and the residue position are given for each sequence. Residues are colored according to their physicochemical properties (aliphatic, salmon; aromatic, orange; positively-charged, blue ; negatively-charged, red ; hydrophilic, green ; proline and glycine residues, magenta ; cysteine residues, yellow).The sequences are ordered following the evolutionary timeline of species and the divergence time is given following molecular clock phylogenetics studies[2]. This phylogenetic analysis identifies three distinct subgroups called Osh6p/Osh7p, ORP5/8 and ORP9/10/11 clade, which would have evolved independently from an ancestral sequence common to Holomycota and Holozoa one billion years ago. A star indicates the most basal lineage of each clade. A Jalview analysis, showing consensus and conservation score at the top of the figure, indicates that an acidic motif (consensus EDDDTDDTEEVDEE) localized at the N-terminus of the lid is well-conserved in the Osh6p/Osh7p clade, in particular in Saccharomycotina (highlighted in grey), and also in ORP9/10/11 clade. **(b)** Surface of the Osh6p's structure (PDB ID: 4PH7) showing the degree of conservation of amino acids in the ORD considering all sequence with the PS-recognizing motif LPTFILE (left) identified in our sequence analysis, or only the Saccharomycotina (right). Cyan-to-purple color scale indicates the degree of conservation. The acidic track and the lid are represented in ribbon and the anionic residues of the D/E–rich motif are shown in a stick mode. Source data are provided as a Source Data file.

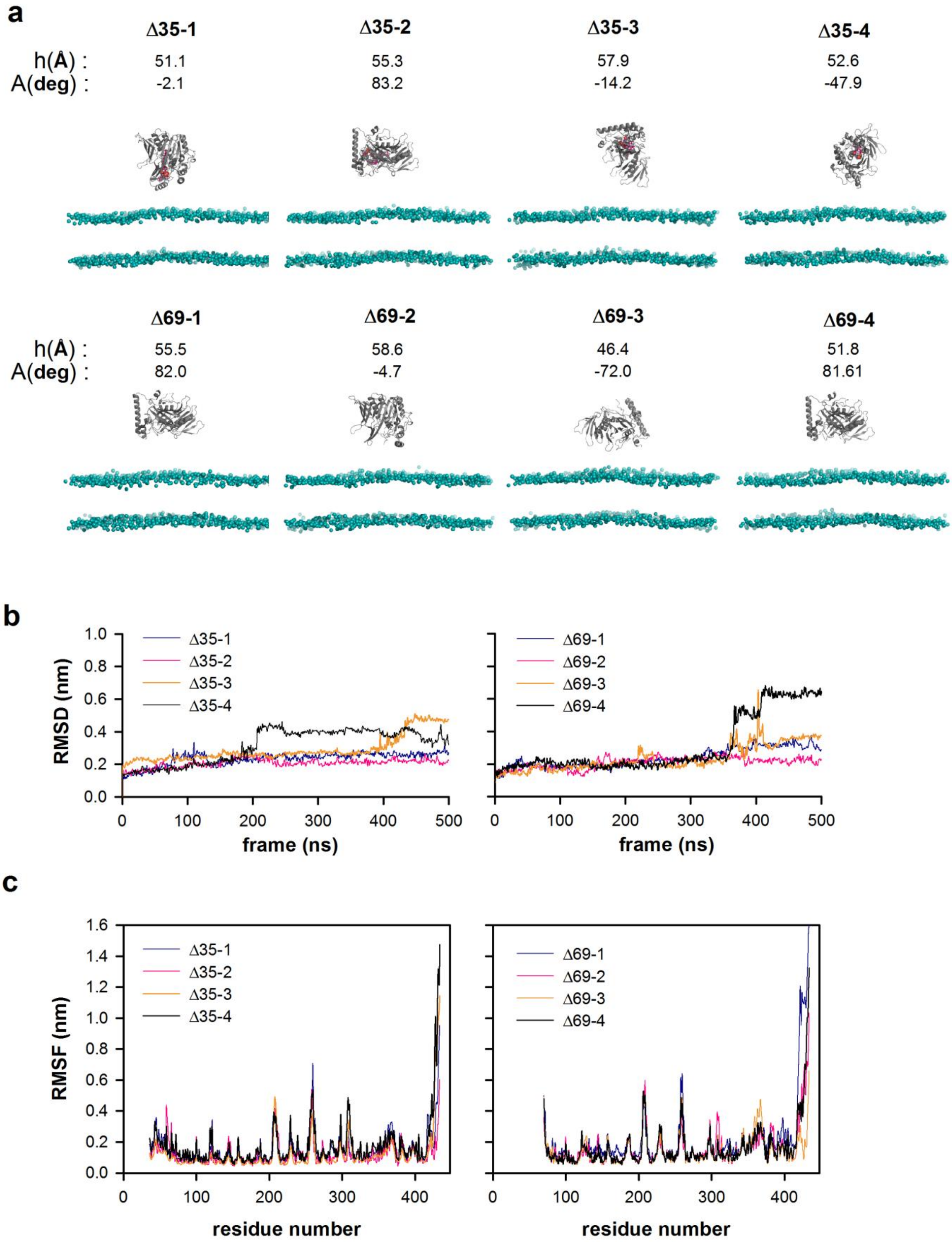

Supplementary Figure 6. RMSD and RMSF profile for Osh6pΔ35 and Osh6pΔ69
(a) Configuration of Osh6pΔ35 and Osh6pΔ69 at the beginning of each trajectory. The initial height (h) between the mass center of each form of Osh6p relative to the plane of the DOPC/POPS bilayer is indicated. The initial angle between the 346-356 segment of the α7 helix and the plane of the membrane is also reported. **(b)** Control plots representing the stability of Osh6pΔ35 (residues 35-432) or Osh6pΔ69 structure (residues 70-432) during four independent 500-ns MD run. The root mean square deviation (RMSD) of the backbone atoms from the equilibrated conformation (0 ns) is presented as a function of time for each trajectory. **(c)** Root mean square fluctuation (RMSF) values of atomic positions computed for the backbone atoms, indicative of the internal protein motion during each MD, are shown as a function of residue number. Source data are provided as a Source Data file.

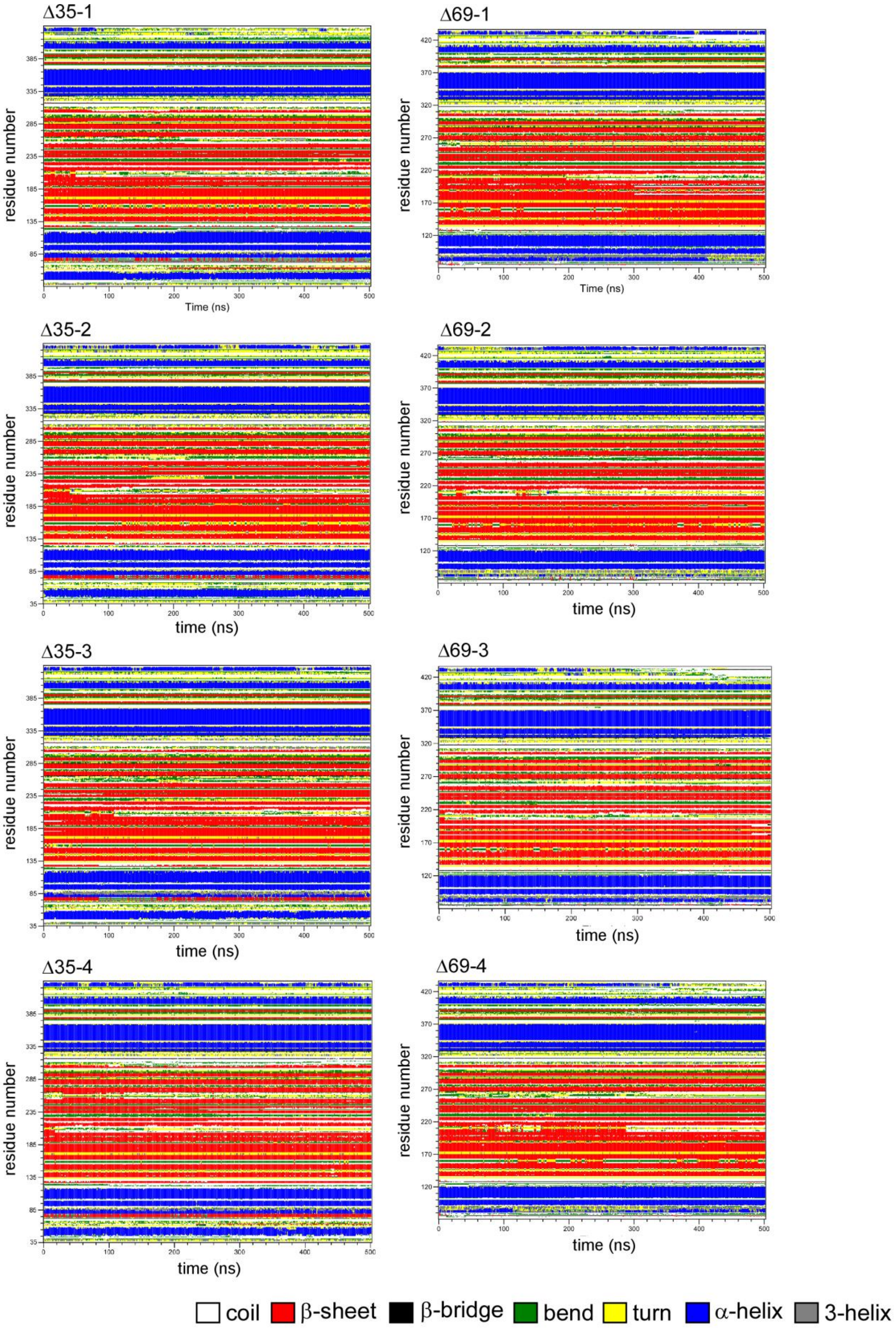

Supplementary Figure 7. Time evolution of the secondary structural elements of Osh6pΔ35 and Osh6pΔ69 along each MD simulation. Osh6pΔ35 (left panel); Osh6pΔ69 (right panel). The x-axis represents the MD trajectory time (in ns), while the residue numbers are shown on the y-axis.

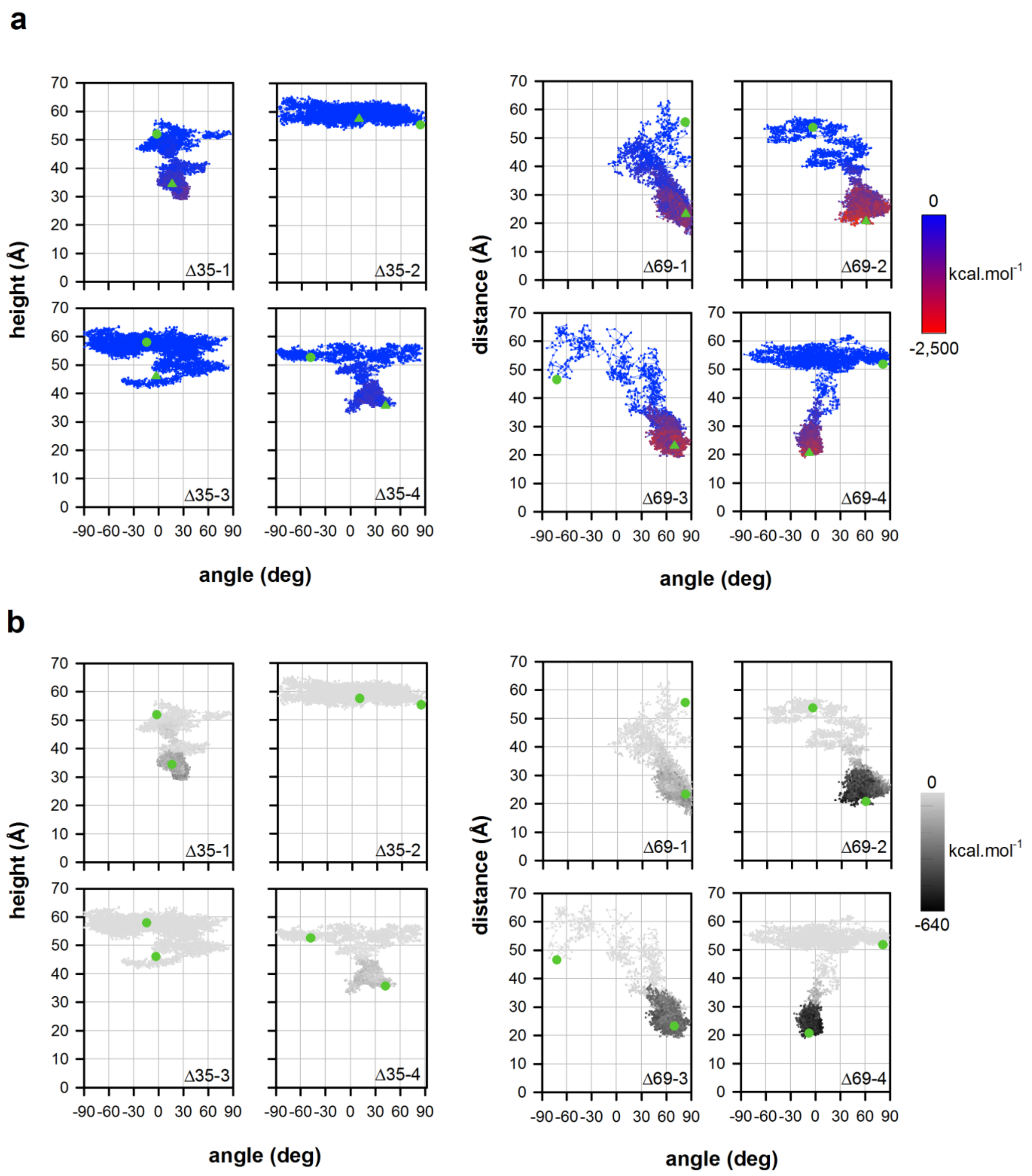

Supplementary Figure 8. Evolution of the protein-membrane interaction energy over time for each trajectory

(a) Co-evolution of the height and orientation of the Osh6pΔ35 or Δ69 structure relative to the membrane during each trajectory is represented as in Figure 5. Each point of the trajectory is coloured according to a code, ranging from blue to red, which is function of the Coulomb energy (from 0 to -2,500 kcal.mol^{-1}). Green circles and triangles correspond to the height and angle values associated to the initial and final configuration (t=500 ns) of each protein, respectively. (**b**) VDW interaction between the protein and the membrane (colour code from light gray to black corresponding to a range of value between 0 and -640 kcal mol^{-1}). Source data are provided as a Source Data file.

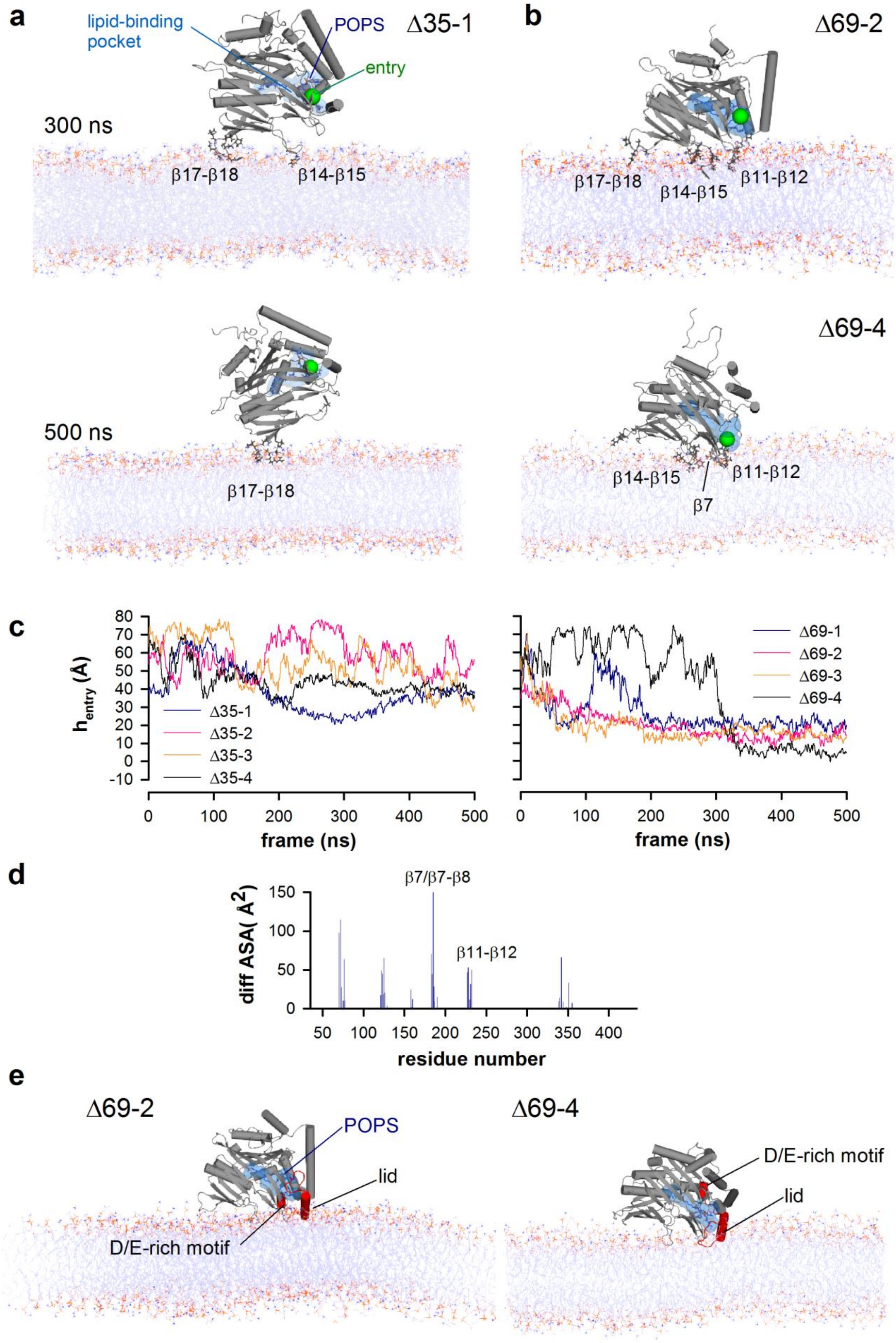

a
lipid-binding pocket
POPS
Δ35-1
entry
300 ns
β17-β18
β14-β15
500 ns
β17-β18
b
Δ69-2
β17-β18
β14-β15
β11-β12
Δ69-4
β14-β15
β11-β12
β7
c
h_entry (Å)
Δ35-1
Δ35-2
Δ35-3
Δ35-4
Δ69-1
Δ69-2
Δ69-3
Δ69-4
frame (ns)
frame (ns)
d
diff ASA(Å2)
β7/β7-β8
β11-β12
residue number
e
Δ69-2
POPS
lid
D/E-rich motif
Δ69-4
D/E-rich motif
lid

Supplementary Figure 9. The lid prevents the lipid-binding pocket from being close to the membrane surface

(a) Configuration of Osh6pΔ35, bound to the surface of the DOPC/POPS (7/3) bilayer at 300 ns (top) and at the end of the Δ35-1 trajectory (bottom). The protein is shown in a cartoon mode and colored in light grey. The side chains of the residues of the β14-β15 and β17-β18 loops are represented in a stick mode and colored in dark grey. The contour of the lipid-binding pocket is represented by a transparent blue surface whereas the center of the pocket entry is represented by a solid green sphere. Atoms of the membrane lipids are shown in a wireframe representation with carbon in light blue, oxygen in red, nitrogen in blue, hydrogen in grey and phosphorus in orange. **(b)** Configuration of Osh6pΔ69, bound to the surface of the lipid bilayer at the end of the Δ69-2 (top) and Δ69-4 trajectories (bottom). The side chains of the residues of the β11-β12, β14-β15 and β17-β18 loops as well as the β7-strand are represented in a stick mode and colored in dark grey. (**c**) Evolution of the height of the pocket entry relative to the plane of the bilayer during 500 ns. **(d)** Difference in accessible surface area (ASA) *per* residue between the Δ35 and Δ69 structures. The position of the residues belonging to the β7-strand, the β7-β8 and the β11-β12 loops, which are unmasked in the absence of the 35-69 region, are indicated. **(e)** The structure of Osh6pΔ35 has been superposed to that of Osh6pΔ69 when bound to the membrane at the end of the Δ69-2 (left) and Δ69-4 trajectories (right). This indicates what would be the theoretical localisation of the lid and the D/E-rich motif if Osh6pΔ35 adopted such a docking geometry on membrane. Likewise, the POPS molecule is represented in a sphere mode to show its position relative to the surface of the bilayer surface. Atoms of the membrane lipids are shown in a wireframe representation with carbon in light blue, oxygen in red, nitrogen in blue, hydrogen in grey and phosphorus in orange. Source data are provided as a Source Data file.

Supplementary Table 1. Primers used to delete region or insert mutations in Osh6p

Deletion/Mutation Designation	Primer name	Sequence
Δ35	Osh6_Δ35_For	CGTGGATCCATGGGCTCCATTGATACCGACGATATTGATGAAGACG
	Osh6_Δ35_Rev	CGTCTTCATCAATATCGTCGGTATCAATGGAGCCCATGGATCCACG
C62S	Osh6_C62S_For	CAATTGAGACCAGGCAGTGATTTGACCAGGATCACC
	Osh6_C62S_Rev	GGTGATCCTGGTCAAATCACTGCCTGGTCTCAATTG
C162S	Osh6_C162S_For	GTCACCATCCTCCAGAAAGTGCATATTTTTACATG
	Osh6_C162S_Rev	CATGTAAAAATATGCACTTTCTGGAGGATGGTGAC
C389S	Osh6_C389S_For	GGCGAAGATCTTGACTATTCTATTTATAAAAATATCCC
	Osh6_C389S_Rev	GGGATATTTTTATAAATAGAATAGTCAAGATCTTCGCC
S190C	Osh6_S190C_For	GATTTTTAGGTAATTCATGTGCGGCCATGATGGATGG
	Osh6_S190C_Rev	CCATCCATCATGGCCGCACATGAATTACCTAAAAATC
T262C	Osh6_T262C_For	GGATATGTTTTTGGATGTTACGATGCAATCGAAGGG
	Osh6_T262C_Rev	CCCTTCGATTGCATCGTAACATCCAAAAACATATCC
L69D[(a)]	Osh6_L69D*_For	GTGATTTGACCAGGATCACCGATCCTACTTTTATTTTAGGG
	Osh6_L69D*_Rev	CCCTAAAATAAAAGTAGGATCGGTGATCCTGGTCAAATCAC
4A	Osh6_4A_For	GCCAATTGATACCGCCGCTATTGCTGCAGACGATGAATCTGG
	Osh6_4A_Rev	CCAGATTCATCGTCTGCAGCAATAGCGGCGGTATCAATTGGC
5A2G	Osh6_5A2G_For	CCGCCGCTATTGCTGCAGGCGGTGCATCTGGTCATAATATTATC
	Osh6_5A2G_Rev	GATAATATTATGACCAGATGCACCGCCTGCAGCAATAGCGGCGG
Δ69	see Moser von Filseck, 2015 [1]	
L69D	see Moser von Filseck, 2015 [1]	
H157A/H158A	see Moser von Filseck, 2015 [1]	

[(a)] These oligonucleotides were designed to introduce the L69D substitution in Osh6p(noC/S190C) bearing the C62S mutation

Supplementary Table 2. Radius *vs* lipid composition of the main liposomes used in the study

Lipid composition (mol/mol)	Hydrodynamic radius ($R_{H,}$ nm)			
	**batch 1**	**batch 2**	**batch 3**	**batch 4**
DOPC (100)	99.9 ± 28.1	118.8 ± 48.4	114.2 ± 39.5$^{(N)}$	105.7 ± 40.3
DOPC/POPS (95/5)	82.4 ± 29.8$^{(N)}$	81.4 ± 28.0$^{(N)}$	93.0 ± 32.0$^{(N)}$	
DOPC/POPS (90/10)	85.4 ± 47.4$^{(N)}$	83.0 ± 25.7$^{(N)}$		
DOPC/POPS (70/30)	100.0 ± 27.0	93.0 ± 32.0	96.4 ± 31.0	
DOPC/POPS (50/50)	95.7 ±37.3$^{(N)}$			
POPS (100)	96.9 ± 27.2$^{(N)}$			
DOPC/POPS/diC16:0-PI4P (66/30/4)	92.0 ± 28.5$^{(N)}$			
DOPC/POPS (98/2)	108.0 ± 40.3			
DOPC/ diC16:0-PI4P (98/2)	100 ± 21.47			
DOPC/diphytanoyl-PS (98/2)	106.8 ± 44.4	93.6 ± 46.7$^{(N)}$	97.6 ± 45.1	
DOPC/diphytanoyl-PS (70/30)	93.6 ± 46.7$^{(N)}$	97.6 ± 45.1$^{(N)}$	89.8 ± 35.9	108.0 ± 44.5
DOPC/diphytanoyl-PS/POPS (70/28/2)	94.6 ± 43.1$^{(N)}$			
DOPC/diphytanoyl-PS/POPS (70/25/5)	83.3 ± 34.6$^{(N)}$			
DOPC/diphytanoyl-PS/POPS (70/25/10)	100.4 ± 35.6$^{(N)}$			
DOPC/diphytanoyl-PS/ diC16:0-PI4P (70/28/2)	99.56 ± 37.8$^{(N)}$			
DOPC/diphytanoyl-PS/ diC16:0-PI4P (70/25/5)	99.9 ± 34.6$^{(N)}$			
DOPC/diphytanoyl-PS/ diC16:0-PI4P (70/25/10)	97.7 ± 26.6$^{(N)}$			
DOPC/diphytanoyl-PC (70/30)	145.0 ± 76.0	107.7 ± 46.5$^{(N)}$		
DOPC/diphytanoyl-PC/diC16:0-PI4P (70/25/5)	95.34 ± 39.38			
DOPC/POPA (70/30)	95.7 ± 27.8$^{(N)}$	97.5 ±40.3$^{(N)}$		
DOPC/POPA/POPS (70/25/5)	98.2 ± 35.2$^{(N)}$			
DOPC/POPA/diC16:0-PI4P (70/25/5)	97.3 ± 41.7$^{(N)}$			
DOPC/PI (70/30)	110.5 ± 45.0$^{(N)}$	97.9 ± 25.7 $^{(N)}$		
DOPC/PI/POPS (70/25/5)	96.1± 39.3$^{(N)}$			
DOPC/PI/diC16:0-PI4P (70/25/5)	116.0 ± 35.6 $^{(N)}$			
DOPC/PI/POPA/POPS (76/15/4/5)	98.3 ± 45.0			
DOPC/ diphyt-PS/PI/POPA/ diC16:0-PI4P (56/25/10/4/5)	98.3 ± 27.8 $^{(R)}$			

$^{(N)}$ liposomes contain trace amount of NBD-PE (0.3 mol%)

$^{(R)}$ liposomes contain 2 mol% of Rhod-PE at the expense of DOPC

Supplementary Table 3. Yeast plasmids used in this study.

Name/Alias	Description	Origin/Reference
**pRS315-Osh6-mCh**	*ADH1pr:OSH6-mCherry,* CEN, *LEU2* (pRS315-based)	Maeda et al, 2013 [3]
**pRS315-Osh6-GFP**	*ADH1pr:OSH6-GFP,* CEN, *LEU2* (pRS315-based)	This study
pAC89 **pOsh6(L69D)**	*ADH1pr:osh6[L69D]-mCherry,* CEN, *LEU2* (pRS315-based)	Moser von Filseck, 2015 [1]
pAC91 **pOsh6(HHAA)**	*ADH1pr:osh6[H157A,H158A]-mCherry,* CEN, *LEU2* (pRS315-based)	Moser von Filseck, 2015 [1]
pAC95 **pOsh6Δ69**	*ADH1pr:osh6[70-448]-mCherry,* CEN, *LEU2* (pRS315-based)	This study
**pSec63-sGFP**	*SEC63-sGFP,* CEN, *URA3* (pRS316-based)	Prinz et al, 2000 [4]
**pC2$_{Lact}$-GFP**	*GPDpr:C2$_{Lact}$-GFP,* CEN, *URA3* (pRS416-based)	Yeung et al, 2008 [5]
pAC100 **pOsh6-mCh-U**	*ADH1pr:OSH6-mCherry,* CEN, *URA3* (pRS315-based)	This study
pAC101 **pOsh6(L69D)-U**	*ADH1pr:osh6[L69D]-mCherry,* CEN, *URA3* (pRS315-based)	This study
pAC102 **pOsh6(HHAA)-U**	*ADH1pr:osh6[H157A,H158A]-mCherry,* CEN, *URA3* (pRS315-based)	This study
pAC103 **pOsh6Δ69-U**	*ADH1pr:osh6[70-448]-mCherry,* CEN, *URA3* (pRS315-based)	This study
pAC104 **pOsh6Δ35-U**	*ADH1pr:osh6[36-448]-mCherry,* CEN, *URA3* (pRS315-based)	This study
pAC106 **pOsh6(5A2G)-U**	*ADH1pr:osh6[D38A,D39A,D41A,E42A,D43G,D44G,E45A]-mCherry,* CEN, *URA3* (pRS315-based)	This study
pAC107 **pC2$_{Lact}$-GFP-L**	*GPDpr:C2$_{Lact}$-GFP,* CEN, *LEU2* (pRS416-based)	This study

Supplementary references

1. Moser von Filseck, J. et al. INTRACELLULAR TRANSPORT. Phosphatidylserine transport by ORP/Osh proteins is driven by phosphatidylinositol 4-phosphate. *Science* **349**, 432-436 (2015).
2. Berbee, M.L. & Taylor, J.W. Dating the molecular clock in fungi – how close are we? *Fungal Biology Reviews* **24**, 1-16 (2010).
3. Maeda, K. et al. Interactome map uncovers phosphatidylserine transport by oxysterol-binding proteins. *Nature* **501**, 257–261 (2013).
4. Prinz, W.A. et al. Mutants affecting the structure of the cortical endoplasmic reticulum in Saccharomyces cerevisiae. *J Cell Biol* **150**, 461-74 (2000).
5. Yeung, T. et al. Membrane phosphatidylserine regulates surface charge and protein localization. *Science* **319**, 210-213 (2008).
