# Supplementary material for: An electrostatic switching mechanism to control the lipid transfer activity of Osh6p
Source: Nat Commun. 2019 Sep 2;10:3926. doi: 10.1038/s41467-019-11780-y (PMC6718676; doi:10.1038/s41467-019-11780-y)
Supplement: Supplementary file 6 — Description of Additional Supplementary Files [file 41467_2019_11780_MOESM6_ESM.pdf]

Title: Supplementary Movie 1.
Description: All-atom MD simulation showing the binding of Osh6pΔ35 to a DOPC/DOPS (70/30) bilayer (Δ35-1trajectory). The length of the simulation is 500 ns. The protein is represented in a ribbon mode with the lid (region [35-69]) coloured in red and the α7 helix in green. The POPS molecule inside the lipid-binding pocket is represented in a sphere mode with carbon in cyan. The side chain of residues that preferentially insert into the membrane is represented in a stick mode with carbon in grey, oxygen in red and nitrogen in blue. Lipids in the membrane are represented in wire mode with carbon in pale blue, oxygen in red, nitrogen in blue and phosphorus in orange. Water molecules are not showed.

Title: Supplementary Movie 2.
Description: The movie corresponds to the Δ35-2 trajectory (500 ns) during which Osh6pΔ35 does not bind to the DOPC/DOPS bilayer.

Title: Supplementary Movie 3.
Description: All-atom 500-ns MD simulation showing the binding of Osh6pΔ69 to a DOPC/DOPS (70/30) bilayer (Δ69-2 trajectory).

Title: Supplementary Movie 4.
Description: All-atom 500-ns MD simulation of Osh6pΔ69 in the presence of a DOPC/DOPS (70/30) bilayer (Δ69-4 trajectory). The protein adopts a docking geometry on the surface of the membrane that is different from the one observed at the end of the Δ69-1 to 3 trajectories.
