# Supplementary material for: An electrostatic switching mechanism to control the lipid transfer activity of Osh6p
Source: Nat Commun. 2019 Sep 2;10:3926. doi: 10.1038/s41467-019-11780-y (PMC6718676; doi:10.1038/s41467-019-11780-y)
Supplement: Supplementary file 7 — Reporting Summary [file 41467_2019_11780_MOESM7_ESM.pdf]

nature research

Corresponding author(s): Guillaume DRIN

Last updated by author(s): 18/07/19

Reporting Summary

Nature Research wishes to improve the reproducibility of the work that we publish. This form provides structure for consistency and transparency in reporting. For further information on Nature Research policies, see Authors & Referees and the Editorial Policy Checklist.

Statistics

For all statistical analyses, confirm that the following items are present in the figure legend, table legend, main text, or Methods section.

n/a	Confirmed	
☐	☒	The exact sample size (*n*) for each experimental group/condition, given as a discrete number and unit of measurement
☐	☒	A statement on whether measurements were taken from distinct samples or whether the same sample was measured repeatedly
☐	☒	The statistical test(s) used AND whether they are one- or two-sided<br>*Only common tests should be described solely by name; describe more complex techniques in the Methods section.*
☒	☐	A description of all covariates tested
☒	☐	A description of any assumptions or corrections, such as tests of normality and adjustment for multiple comparisons
☐	☒	A full description of the statistical parameters including central tendency (e.g. means) or other basic estimates (e.g. regression coefficient) AND variation (e.g. standard deviation) or associated estimates of uncertainty (e.g. confidence intervals)
☐	☒	For null hypothesis testing, the test statistic (e.g. *F*, *t*, *r*) with confidence intervals, effect sizes, degrees of freedom and *P* value noted<br>*Give P values as exact values whenever suitable.*
☒	☐	For Bayesian analysis, information on the choice of priors and Markov chain Monte Carlo settings
☒	☐	For hierarchical and complex designs, identification of the appropriate level for tests and full reporting of outcomes
☒	☐	Estimates of effect sizes (e.g. Cohen's *d*, Pearson's *r*), indicating how they were calculated

Our web collection on statistics for biologists contains articles on many of the points above.

Software and code

Policy information about availability of computer code

Data collection	Commercial PANORAMA software for fluorescence data acquired with the Shimadzu RF 5301-PC ; Commercial Control 1.10 for data acquisition from the fluorescence reader TECAN M1000 Pro ; Commercial Fusion Capt Advance FXS for gel imager Fusion FX ; commercial Metamorph 7 sofware for acquisition of cell image by confocal microscopy ; academic GROMACS 5.0 for performing MD simulations ; commercial NACCESS for ASA calculation ; PyMOL + APBS plugin for electrostatic potential map calculations, commercial SpectraManager for acquisition of circular dichroism data with Jasco J-815
Data analysis	Commercial AIDA/1D for gel quantification ; commercial Excel for analysing fluorescence raw data ; academic DICHROWEB software for CD spectra analysis, commercial PyMOL and academic VMD for molecular vizualization ; academic MD analysis package for trajectory analysis ; iacademic GROMACS 5.0 package for classical MD analysis (secondary structure, RMSD, RMSF) ; in-house Python scripts for analysis of distance and angle in MD trajectories ; academic Jalview Version 2 for multiple sequence alignment and analysis ; On line sofware for Mann-Whitney test (https://ccb-compute2.cs.uni-saarland.de/wtest/)

For manuscripts utilizing custom algorithms or software that are central to the research but not yet described in published literature, software must be made available to editors/reviewers. We strongly encourage code deposition in a community repository (e.g. GitHub). See the Nature Research guidelines for submitting code & software for further information.

Data

Policy information about availability of data

All manuscripts must include a data availability statement. This statement should provide the following information, where applicable:

- Accession codes, unique identifiers, or web links for publicly available datasets
- A list of figures that have associated raw data
- A description of any restrictions on data availability

Data supporting the findings of this manuscript are available from the corresponding author upon reasonable request.

Field-specific reporting

Please select the one below that is the best fit for your research. If you are not sure, read the appropriate sections before making your selection.

☒ Life sciences ☐ Behavioural & social sciences ☐ Ecological, evolutionary & environmental sciences

For a reference copy of the document with all sections, see nature.com/documents/nr-reporting-summary-flat.pdf

Life sciences study design

All studies must disclose on these points even when the disclosure is negative.

Sample size	In quantitative in vitro biochemical/biophysical experiments, sample size was chosen according to the type of assays. For fluorescence-based assays, as we work in very controlled in vitro systems with pure proteins and robust signal, a minimal number of three independent assays is generally enough to get the basic estimator, Mean and S.E.M. to unambiguously discriminate between different experimental conditions (mutation , difference in lipid composition). In several cases, for flotation assays that are more subject to experiment variations, or when a large amount of material (protein) is available or if the assay is semi-automatized (i.e., using a fluorescence plate reader), the number of experiment has been increased such as to perform, if relevant, a Mann–Whitney test which is a non-parametric statistical U-test perfectly adapted to test the H0 hypothesis on two small subsets of data (with one set including at least n=3 values and a second set with at least n=4 values). For fluorescent microscopy experiments in yeast, representative images from at least two independent experiments are shown, with >50 cells imaged per experiment. Fluorescent time-course assays were repeated 3 or more times with independent transformants, with >50 cells images per experiment. Yeast growth assays were repeated at least 3 times. For MD simulations, to compare two different systems, n=4 independent trajectories of 500 ns have been performed, which is considered to be appropriate in this field.
Data exclusions	In fluorescence assays, a few data has been excluded as there was a doubt on the quality of measured signal due to the bleaching/oxidation of our fluorescent reagents (i.e. labeled proteins, even maintained on ice and in the dark, tends to be less bright at the end of the day), or liposomes preparation issues. In this case, a novel experiment is performed in the same condition with either a new batch of liposomes or a new defrosted probe. For SDS-PAGE analyses, gels showing problems of migration or staining (very twisted band, weak band, high background) preventing correct signal integration when using our image analysis software, were not considered and samples were loaded into a new gel.
Replication	Replication of biochemical experiments has been performed in a systematic manner by applying the same protocol (same temperature, buffer, concentration, data acquisition) but at different days, starting from a new batch of liposomes and in some cases, from different batches of protein using each time fresh material (new defrosted buffer, new defrosted protein aliquot, ..) For some experiments, replications have been done with a distance of several months by two different persons, giving the same result. Replication in MD simulation was achieved by launching distinct trajectories with different initial conditions and velocities.
Randomization	Randomization is not relevant for this study. Samples of proteins and liposomes or cells are representative of the same stock population in the same buffer/medium and temperature condition.
Blinding	Blinding is not relevant to this study because it is technically barely feasible to do not know the molecular composition of each assay.

Reporting for specific materials, systems and methods

We require information from authors about some types of materials, experimental systems and methods used in many studies. Here, indicate whether each material, system or method listed is relevant to your study. If you are not sure if a list item applies to your research, read the appropriate section before selecting a response.

Materials & experimental systems

n/a	Involved in the study	
☒	☐	Antibodies
☐	☒	Eukaryotic cell lines
☒	☐	Palaeontology
☒	☐	Animals and other organisms
☒	☐	Human research participants
☒	☐	Clinical data

Methods

n/a	Involved in the study	
☒	☐	ChIP-seq
☒	☐	Flow cytometry
☒	☐	MRI-based neuroimaging

Eukaryotic cell lines

Policy information about cell lines

Cell line source(s)	The origin of yeast and bacterial strains is given in the Methods section
Authentication	*Describe the authentication procedures for each cell line used OR declare that none of the cell lines used were authenticated.*

Mycoplasma contamination	*Confirm that all cell lines tested negative for mycoplasma contamination OR describe the results of the testing for mycoplasma contamination OR declare that the cell lines were not tested for mycoplasma contamination.*
Commonly misidentified lines<br>(See ICLAC register)	*Name any commonly misidentified cell lines used in the study and provide a rationale for their use.*
